# Influence of Interface Type on Dynamic Deformation Behavior of 3D-Printed Heterogeneous Titanium Alloy Materials

**DOI:** 10.3390/ma17081922

**Published:** 2024-04-22

**Authors:** Anmi Li, Yumeng Luo, Boya Wang, Xiaoyun Song

**Affiliations:** 1 State Key Laboratory of Nonferrous Metals and Processes, China GRINM Group Co., Ltd., Beijing 100088, China; lam19125@outlook.com (A.L.); boya0314@hotmail.com (B.W.); songxy@grinm.com (X.S.); 2 GRIMAT Engineering Institute Co., Ltd., Beijing 101407, China; 3 General Research Institute for Nonferrous Metals, Beijing 100088, China

**Keywords:** high strain rate, shear band, deformation mechanism, dynamic fracture

## Abstract

Using the Split Hopkinson Pressure Bar technique, strain-limited dynamic compressive loading experiments were performed on TA1/TA15 heterostructure (HS) materials. The plastic deformation mechanisms, fracture forms, and energy absorption properties of an HS material with a metallurgical bonding interface (MB) and an HS material without a metallurgical bonding interface (NMB) are compared and analyzed. The results show that there is no significant difference between the two deformation mechanisms. The fracture forms are all “V-shaped” fractures within the TA1 part. The NMB was carried for 57 μs before failure and absorbed 441 J/cm^3^ of energy. The MB was carried for 72 μs before failure and absorbed 495 J/cm^3^ of energy. Microstructure observations show that there is a coordinated deformation effect near the MB interface compared to the NMB, with both TA1 and TA15 near the interface carrying stresses. This causes an enhancement of the MB load-bearing time and a 12% increase in energy absorption.

## 1. Introduction

Titanium alloys have an excellent dynamic deformation ability [1]. Therefore, titanium alloys have a large number of applications in the fields of armor, aerospace, and vehicles that are resistant to high-speed impacts [2,3,4,5,6]. At this stage, the enhancement of the resistance of homogeneous materials to high-velocity impacts by conventional means is at its upper limit.

HS materials are a group of materials that are composites of multiple materials with different mechanical or physical properties. For HS materials, the interactions between these hetero-zones produce synergistic effects. Some studies have shown that some mechanical properties of HS materials can exceed the properties of homogeneous materials in HS materials [7]. Due to the plastic incompatibility between the hard and soft zones in HS materials [8], geometrically necessary dislocations (GNDs) in the soft zones pile up and accumulate near the zone boundaries, generating back stress in the soft zones and positive stress in the hard zones, which collectively produce heterogeneous deformation-induced (HDI) stresses, thereby increasing the strength and ductility of the material [9,10,11]. HS materials [12] include heterogeneous lamellar structured materials [13], gradient structured materials [14,15,16,17,18], lamellar structured materials [19,20,21], and bimodal/multimodal structures [22,23]. HS materials without metallurgical bonding interfaces have been applied in the field of armor, such as ceramic/metal composite armor, which has attracted a lot of attention for its excellent ballistic performance [24,25].

Miao et al. [26] investigated Ti/Al_3_Ti HS materials with different residual Al contents and found that the elongation of Ti/Al_3_Ti/Al was 64.2~228.5% higher than that of Ti/Al3Ti. Fan et al. [27] produced TiC/(TC18 + TC4) composites with HS materials. Compared to the homogeneous materials, they found that the HS materials have higher strength and ductility (fracture strength of 1150 MPa and uniform elongation of 5%). Jiang et al. [28] manufactured TC4-Nb-NiTi alloy components using multi-wire arc additive manufacturing and tested their microhardness (393 HV) and ultimate compressive strength (1420 MPa). Currently, for HS materials, researchers mainly focus on their mechanical properties under quasi-static conditions [29,30,31,32,33,34]. There are fewer studies on the dynamic deformation behavior and microstructure evolution of HS materials under high-speed-impact conditions [19,20]. He et al. [35] characterized the dynamic deformation behavior of HS materials of low-carbon steel/304 stainless steel at high strain rates and found that the difference in hardness between the two components in HS materials is an important factor influencing the extension of the adiabatic shear band (ASB). However, the study is not relevant to titanium alloys. Guo et al. [36] prepared an HS material from CP-Ti/TC4 using explosive welding and performed dynamic compression tests on it, and found that CP-Ti always undergoes destruction before TC4. However, Guo et al. did not elaborate on the deformation mechanism and energy absorption properties of HS materials.

Strain-rate strengthening effects, inertia effects, and thermal softening effects all affect the plastic deformation mechanisms and fracture forms of materials [35,37,38,39,40]. Therefore, although some results of mechanical properties under quasi-static conditions have been available for HS materials, it is still impossible to predict their dynamic deformation behavior simply from the deformation behavior under quasi-static conditions.

There is a lack of studies on the dynamic deformation behavior of HS titanium alloy materials at high strain rates. In this study, HS titanium alloy materials with good interfacial bonding were prepared by additive manufacturing technology. The fracture morphology, deformation mechanism, and energy-absorbing properties of HS materials are discussed in detail with respect to the influence of metallurgical interfaces on the dynamic deformation behavior of HS materials. The materials and methods used in this experiment are described in Section 2 of the paper. Experimental results on the microstructure and properties of HS materials before deformation occur are given in the first part of Section 3 of this paper. The dynamic mechanical properties of HS materials are discussed in part 2 of Section 3, and the energy absorbed by HS materials before failure is calculated by derivation of the equation. In part 3 of Section 3, the microstructure states of HS materials at different locations after dynamic deformation are demonstrated by SEM and EBSD methods, and the failure forms and deformation mechanisms of HS materials are discussed and analyzed. Finally, conclusions are reported in Section 4.

## 2. Materials and Methods

**Figure 1:**
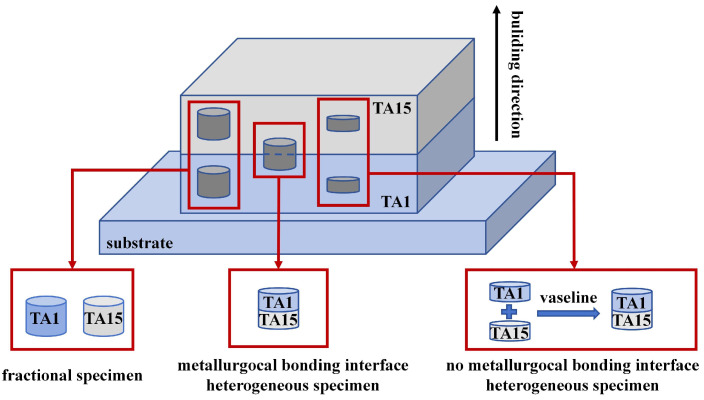
Schematic of 3D-printed TA1/TA15 heterogeneous titanium alloy layered material.

In this study, TA1 (CP-Ti) and TA15 (Ti-6.5Al-1Mo-1V-2Zr) powders with particle sizes ranging from 53 to 150 μm were purchased from GRIMAT Additive Manufacturing Technology Co., Ltd. (Beijing, China). The chemical composition of the powder is shown in Table 1. HS materials of TA1/TA15 were prepared using laser-directed energy deposition, which is shown schematically in Figure 1. The molding process parameters are as follows: the laser power is 1500 W, the scanning rate is 10 mm/s, and the layer thickness is 0.6 mm, in which the protective gas and the carrier gas for powder delivery are high-purity argon. From the TA1 part 5 mm from the interface, 30 g of debris and three small cylinders measuring Φ3 × 20 mm were taken. From the TA15 part 5 mm from the interface, 30 g of debris and three small cylinders measuring Φ3 × 20 mm were taken. Using an Inductively Coupled Plasma Spectrometer, the chemical compositions of TA1 and TA15 at 5 mm from the interface were analyzed, and the test results are listed in Table 1.

Three types of specimens were used in this study. The sampling method is shown in Figure 1. The Φ5 × 5 mm cylindrical specimen was obtained at the interfaces of the HS as the heterogeneous specimen with a metallurgical bonding interface, abbreviated as MB. The non-metallurgical bonding heterogeneous specimen was formed by adding petroleum jelly to the centers of two homogeneous cylindrical specimens Φ5 × 2.5 mm, abbreviated as NMB. The homogeneous Φ5 × 5 mm cylindrical specimen was made as the control specimen. The three types of specimens were cut along the longitudinal section by a DK7735 wire-cutting machine (Taizhou Terui CNC Machine Co., Ltd., Taizhou, China). The longitudinal section of the specimen was ground to 5000 mesh. Electrolytic polishing (voltage range 56–62 V, time range 13–15 s, current range 0.16–0.24 A) was carried out using 5 vol.% HClO_4_ + 95 vol.% CH_3_COOH solution to obtain electron backscatter diffraction (EBSD) specimens. Scanning electron microscopy (SEM) specimens were obtained by etching in a mixture of 1HF:2HNO_3_:20H_2_O solution for 7–8 s. The microstructure of the HS materials was characterized by a JSM-7900 scanning electron microscope (JEOL Ltd., Tokyo, Japan) equipped with an EBSD probe.

**Figure 2:**
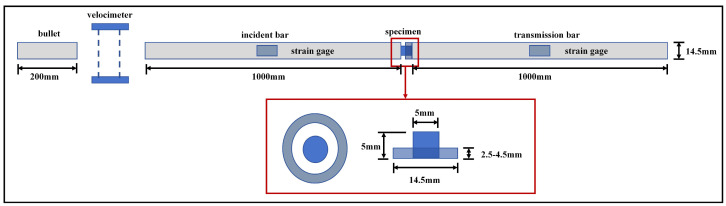
Schematic diagram of SHPB. The red box shows how the stopper ring is assembled and its dimensions.

The dynamic properties of the materials were tested on a Split Hopkinson Pressure Bar Testing Machine (SHPB). The schematic diagram of the SHPB setup is shown in Figure 2. Different height limit rings were utilized to bring the macroscopic deformation of the specimen to a set value in order to obtain a heterogeneous specimen in the critical damage state, where the top and left views of the stopper ring and the specimen are shown in Figure 2.

## 3. Results and Discussion

### 3.1. Microstructure of Heterogeneous Material TA1/TA15 before Deformation

**Figure 3:**
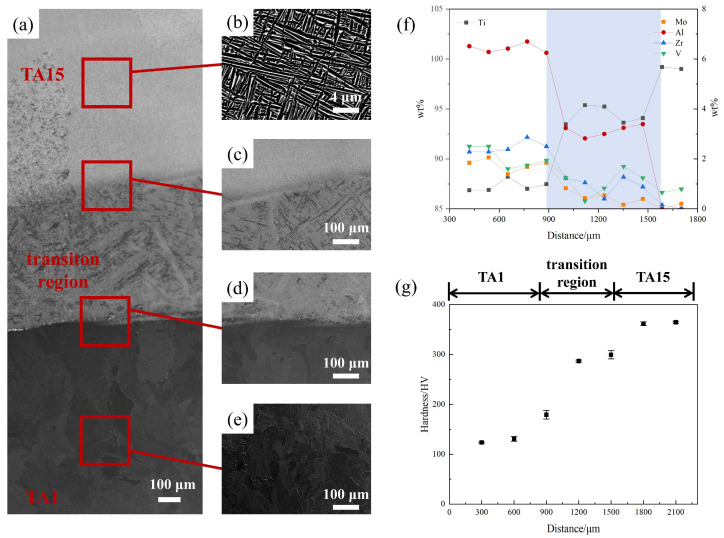
Microstructure, composition distribution, and hardness variation near the interfaces before deformation: (**a**–**e**) SEM images near the interfaces of the HS materials before deformation; (**f**) composition distribution near the interfaces of the HS materials before deformation; (**g**) hardness variation near the interfaces of the HS materials before deformation.

The microstructure at the interface of the TA1/TA15 HS material is shown in Figure 3a–e. The microstructure of the TA1 part consists of irregularly shaped α-phase blocks with a relatively large grain size, approximately 80 μm. The TA15 part is composed of a staggered longitudinal acicular α-phase, where the acicular grains have an average width of about 0.90 μm with an aspect ratio of 19. In the transition region, there exists a coarser α-phase structure, with an average width of 7 μm and an aspect ratio of 16.

In order to analyze the composition and properties of the transition region deeply, energy-dispersive X-ray spectrometry (EDS) testing was performed using a JSM-7900 scanning electron microscope. An HV5 microhardness test (test force of 49.03 N, loading time of 10 s) was performed using a WILSON VH1150 Vickers hardness tester (Buehler, Lake Bluff, IL, USA). Figure 3f shows the compositional changes in each element at the interface. The five elements of Ti, Mo, Al, Zr, and V have more apparent changes at the interface, and the width of the interfacial transition region is about 675 μm. In addition, in the transition region at the interface of the HS material and the homogeneous region at 900 μm and 1575 μm, there is a sudden change in the composition, but the internal composition of the transition region changes less. Figure 3g shows the hardness variation near the TA1/TA15 HS material’s interface. Each point in the figure represents the average value of microhardness at three different points at that distance. It can be found that the hardness variation fluctuates less in the TA1 and TA15 parts, where the microhardness of the TA1 part is about 125 HV and the microhardness of the TA15 part is about 360 HV. The transition region’s microstructure hardness is between the microhardness of TA1 and TA15. This is related to the changes in the grain size and chemical composition of the HS material.

### 3.2. Dynamic Mechanical Properties of TA1/TA15

**Figure 4:**
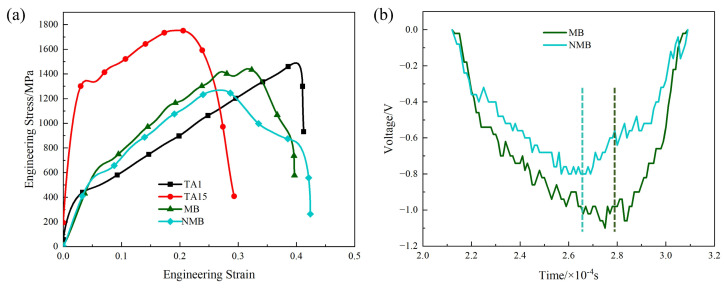
Engineering stress–strain curves and voltage–time curves for HS materials: (**a**) engineering stress–strain curves for MB, NMB, and homogeneous materials; (**b**) voltage–time curves for transmitted waves after dynamic compression of MB and NMB.

Uniaxial dynamic compression tests on HS materials with or without metallurgically bonded interfaces for the TA1 part of the load were first performed, and the engineering stress–strain curves and voltage–time curves were obtained, as shown in Figure 4.

The comparative analysis of the dynamic deformation curves of the heterogeneous and homogeneous materials reveals that the engineering stress–strain curves of the HS materials MB and NMB and the homogeneous material TA1 are close to coinciding when the strain variable is less than 0.04, which indicates that, at this time, TA1 is mainly carrying the deformation in the heterogeneous material. When the strain of the heterogeneous material is more significant than 0.04, the engineering stress–strain curve of the HS materials is between that of the TA15 homogenous materials and that of the TA1 homogenous materials, which suggests that the TA1 portion of the HS materials and the TA15 portion of the HS materials are jointly carrying the deformation. The rheological stress of the NMB starts to decrease when the strain variable is 0.26. At this time, the rheological stress of the MB is still increasing. It does not begin to fall until the strain variable reaches 0.32, indicating that the NMB undergoes stress collapse at the highest point of the rheological stress at an earlier time compared to the MB. Observing the voltage–time curves, it is found that the load-bearing time before failure is 57 μs for the NMB and 72 μs for the MB, which indicates that the load-bearing time before failure is shorter for the NMB and the material has a worse load-bearing capacity at high strain rates.

Since the material studied in this experiment is a heterogeneous titanium alloy layered material, the force and deformation processes of the specimen are not uniform. To more accurately describe the dynamic deformation behavior of the heterogeneous titanium alloy layered material, the energy absorbed by the HS material is used as a characterizing parameter to evaluate the anti-high-speed impact-bearing effect of the two materials. As energy is conserved in the dynamic deformation process of HS materials, the energy incident in the elastic bar can be divided into three parts: the energy reflected in the elastic bar, the energy transmitted in the elastic bar, and the energy absorbed by the specimen. Therefore, the energy absorbed by the specimen can be expressed by the following formula based on these three forms of energy, specifically Equation (1):

(1) $$E_{S}{=E}_{I}-E_{R}-E_{T}$$

where $E_{S}$ is the energy absorbed by the specimen, $E_{I}$ is the incident energy, $E_{R}$ is the reflected energy, and $E_{T}$ is the transmitted energy.

$E_{I}$, $E_{R}$, and $E_{T}$ are the energies carried by the stress wave in the elastic bar, and $E_{B}$ is the energy carried by the stress wave in the elastic bar; the details are shown in Equation (2):

(2) $$E_{B}{=\mathrm{AE}}_{b}\int_{0}^{L}\varepsilon^{2}\mathrm{dx}{=\mathrm{AE}}_{b}c_{0}\int_{t_{1}}^{t_{2}}\varepsilon^{2}\mathrm{dt}$$

where A denotes the cross-sectional area of the bar, $E_{b}$ denotes the modulus of elasticity of the bar, L denotes the length of a complete stress wave propagating through the bar, ε denotes the strain of the bar, $c_{0}$ denotes the wave speed of the bar, $t_{1}$ denotes the beginning of the propagation of the stress wave recorded by the strain gauges affixed to the bar, and $t_{2}$ denotes the end of the stress wave propagation recorded by the strain gauges affixed to the bar.

**Figure 5:**
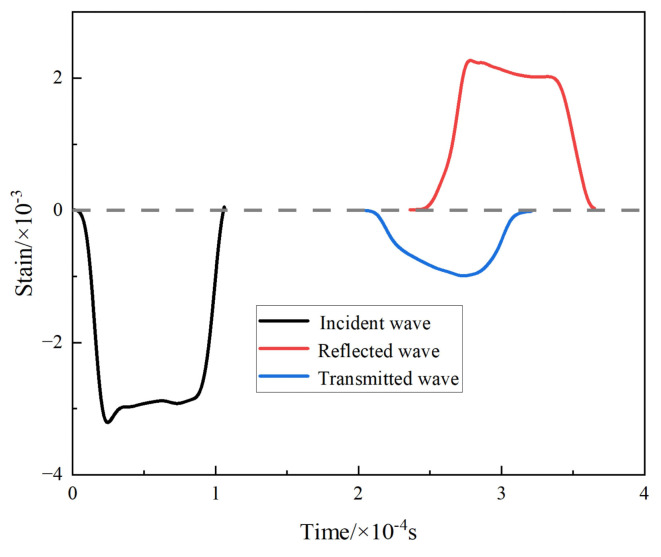
Strain–time curves for incident, reflected, and transmitted waves.

According to the data recorded by the strain gauges, the strain–time curves of the incident wave, reflected wave, and transmitted wave can be derived, and the specific curves are shown in Figure 5. Substituting the curves into Equation (2), we can obtain $E_{I}$, $E_{R}$, and $E_{T}$.

According to the above method to calculate the energy absorbed per unit volume of the material, the energy absorbed per unit volume before the destruction of the NMB is 441 J/cm^3^, and the energy absorbed per unit volume before MB is 495 J/cm^3^. Compared with the NMB, the energy absorbed during the dynamic deformation of the MB is much higher, with a year-on-year increase of about 12%. It indicates that the MB can better resist the damage caused by adiabatic shear.

### 3.3. Microstructure State of TA1/TA15 after Deformation

**Figure 6:**
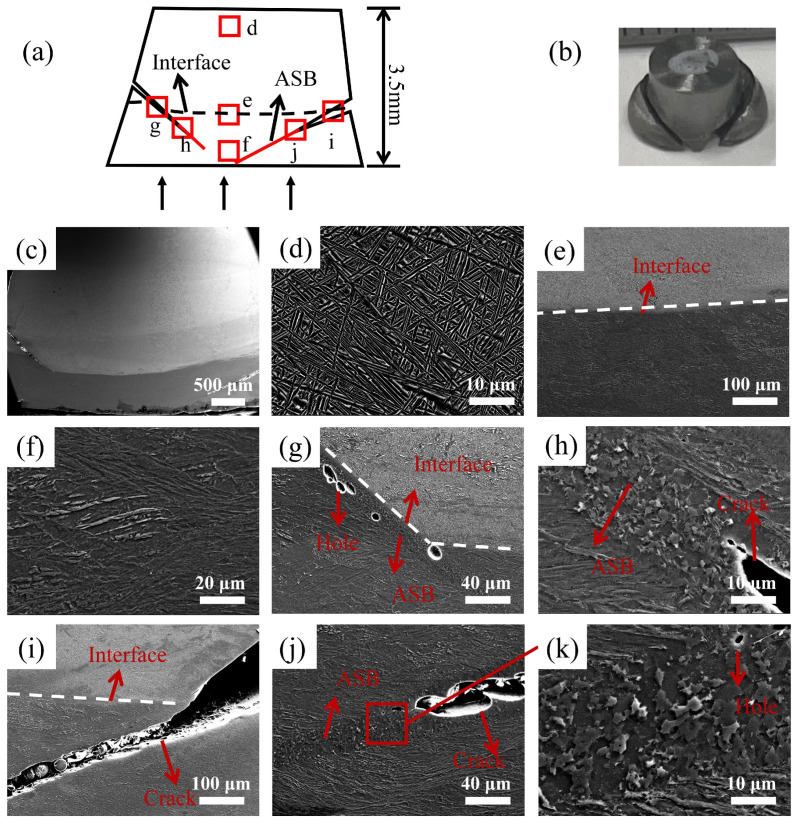
Microstructure of each part after dynamic deformation of MB with a deformation amount of 0.3: (**a**) schematic of longitudinal section after dynamic deformation of MB; (**b**) macroscopic morphology after dynamic deformation of MB; (**c**) microscopic morphology after dynamic deformation of MB in longitudinal section; (**d**–**j**) SEM of the position of the red square in schematic (**a**); (**k**) microstructure of the ASB after magnification in (**j**).

**Figure 7:**
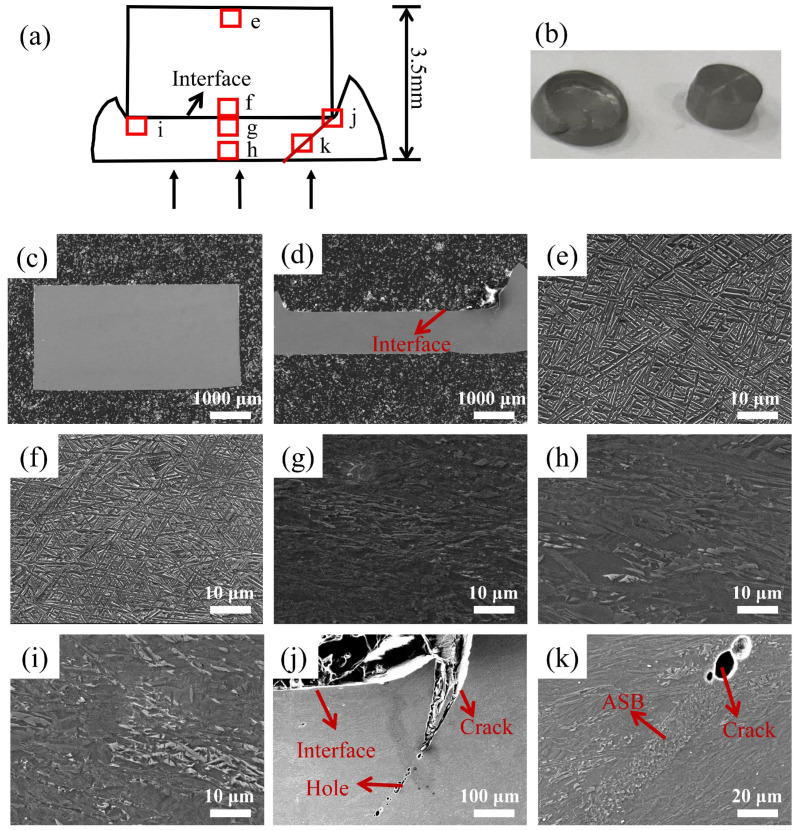
Microstructure of each part after dynamic deformation of NMB with a deformation amount of 0.3: (**a**) schematic of longitudinal section after dynamic deformation of NMB; (**b**) macroscopic morphology after dynamic deformation of NMB; (**c**) micromorphology of the longitudinal section of part of TA15 after dynamic deformation of the NMB; (**d**) micromorphology of the longitudinal section of part of TA1 after dynamic deformation of the NMB; (**e**–**k**) SEM of the position of the red square in schematic (**a**).

In order to explore the microstructure evolution law and dynamic failure mode of the heterogeneous specimen with or without a metallurgical bond interface, combined with the engineering stress–strain curve analysis, a stopper ring is used to control the strain of TA1/TA15 HS materials so that the material is in the critical damage state. The microstructure of each specimen region in the crucial damage state is compared and analyzed by SEM, and the macroscopic morphology and microstructure of the two interface types of HS materials after dynamic deformation are shown in Figure 6 and Figure 7.

Figure 6 shows the microstructure of each region of the longitudinal section of the specimen after dynamic compression of the MB with a strain factor of 0.3. Among them, Figure 6a–c show the schematic diagram of the longitudinal section of the specimen, the macroscopic morphology, and the microstructure morphology after deformation. From the figure, it can be seen that the deformation of the TA15 part of the MB is small, the TA1 part has developed microcracks at the edge of the interface, the interface is protruding toward the TA1 part, and the interface at the edge is at approximately 45° from the loading direction. This indicates that during the dynamic deformation of the MB, the ASB starts to sprout from the interface of TA1 and TA15, and expands by 45° in the region of TA1, eventually forming a “V-shaped” crack.

Figure 6d–f show the schematic diagram of the microstructure near the surface of TA15, at the interface, and near the surface of TA1. Compared with before deformation, the microstructural change in TA15 is not apparent, and there are more elongated grains in TA1, which is very different from before deformation, indicating that TA1 partially carries most of the deformation. Figure 6g–j show the microstructures of the ASB and cracks in Figure 6a. The internal microstructure of the ASB is broken into fine equiaxed grains, and the TA1 matrix portion on the outside of the ASB shows elongated grains along the direction of the ASB. Observing the microstructure of the crack tip, it is found that there are ASBs and small holes along the extension direction of the crack tip, and with the increase in the strain, the tiny holes will gradually increase until they are connected to the crack tip, making the crack continue to extend along the direction of the ASB.

Figure 7 shows the microstructure of each region of the longitudinal section of the specimen after dynamic compression of the NMB with a strain of 0.3. Among them, Figure 7a–d show the schematic diagram of the longitudinal section of the specimen, the macroscopic morphology, and the histomorphology after deformation, respectively. As can be seen from the figure, with the increase in deformation, the part of TA15 in the NMB is undeformed. It remains cylindrical, and the part of TA1 is deformed more, and the macroscopic morphology shows that its two sides bulge upward and wrap TA15. Figure 7e–h show the microstructures near the surface of TA15, at the interface, and near the surface of TA1, represented in the schematic in Figure 7a, respectively. The changes in the microstructure of TA15 are not obvious, and there are more fine grains in the TA1 part, which indicates that the deformation of the TA1 part is larger. Figure 7i–k show the microstructure at the interface edge, and it was found that the cracks sprouted from the junction of the TA15 edge and the interface, and expanded towards the TA1 portion along the 45° direction until they completely penetrated the TA1 material. Due to the absence of a metallurgical bonding interface for the NMB, there is no coordinated deformation effect near the interface. The deformation process does not deform the TA15 material at the edge of the interface with the larger region of deformation of TA1 shear. With the increase in the deformation, the region appeared to have a concentration of stress and a growth of the ASB within the TA1.

**Figure 8:**
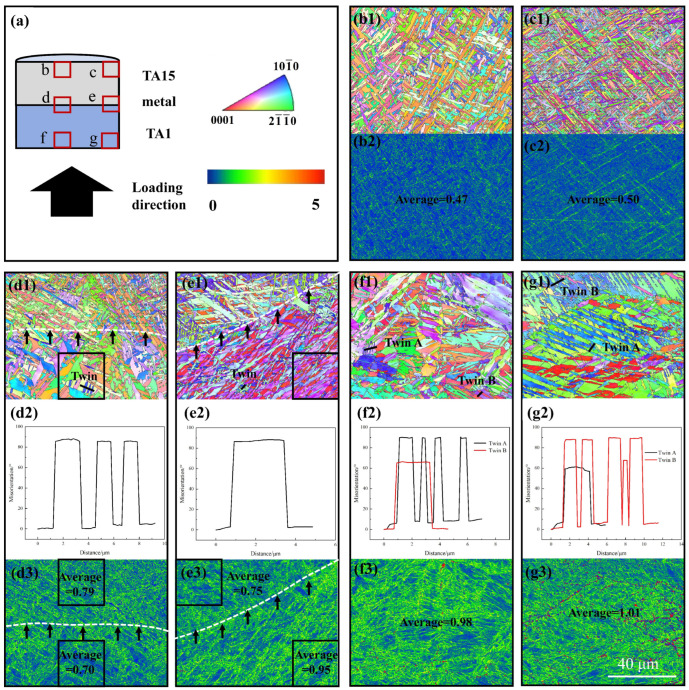
Grain orientation maps and dislocation density distribution cloud maps at various locations after dynamic deformation of MB with a strain of 0.28: (**a**) Schematic diagram of MB loading, with scales of polar and KAM maps of IPF. (**b1**,**c1**,**d1**,**e1**,**f1**,**g1**) IPF maps at the red square position in schematic (**a**), where the white dashed lines are the interfaces. (**b2**,**c2**,**d3**,**e3**,**f3**,**g3**) are the red square location KAM plots, where the mean value of KAM has been calculated and is labeled in the KAM plots; since the positions of (**d3**,**e3**) are in the vicinity of the interface, it is necessary to calculate the average value of KAM on both sides of the interface, which is calculated within the black square in the KAM plot. (**d2**,**e2**,**f2**,**g2**) show the orientation difference plots of the corresponding twins, where the characteristic corners have been labeled in the IPF plots of (**d1**,**e1**,**f1**,**g1**).

EBSD obtained the IPF and KAM maps by observing each part of the specimen before adiabatic shear damage occurred in HS and was used to analyze the degree of deformation concentration in the HS before adiabatic shear damage occurred. Figure 8 shows the EBSD results of the longitudinal section of the specimen after dynamic compression of the MB. Figure 8a shows the MB loading schematic, and Figure 8(b1–g3) shows the IPF and KAM maps at the red box location of the schematic. Observing Figure 8(b1–c2), it is found that the microstructure of the MB near the surface of TA15 does not change much, and the average values of KAM are all smaller, which indicates that the stress concentration at this location is low and carries less stress.

Observation of the center position of the MB’s interface, see Figure 8(d1), reveals that the interface is in the horizontal direction, with a TA1 microstructure below the interface, and the presence of some twins, which are $\left\{10\bar{1}2\right\}$-type twins according to the analysis of the characteristic angles in Figure 8(d2). Since there are two kinds of microstructures, TA1 and TA15, in the position of Figure 8(d3), to compare the stresses of the two microstructures, the average values of KAM of TA15 microstructure above the interface and TA1 microstructure below the interface are calculated, respectively. It is found that the difference between the average values is not large, but it is larger than the average value near the surface of TA15, which indicates that the stress concentrations of TA15 and TA1 at the center of the interface are comparable, and they are larger than that near the surface of TA15. This indicates that both the TA15 and TA1 at the center of the interface carry part of the stress during the dynamic deformation process.

**Figure 9:**
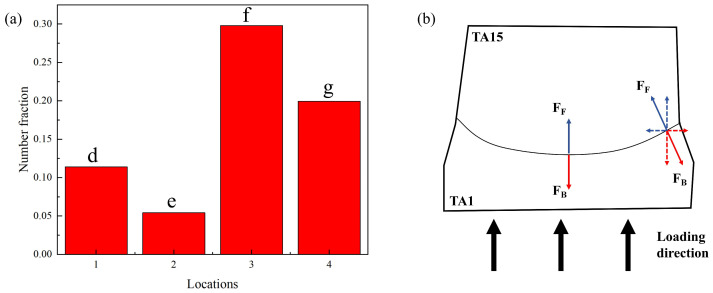
(**a**) Histogram of the twin density at the position of Figure 8(d1,e1,f1,g1), where Figure 8(d1,e1) are in the vicinity of the interface, and only the twin density of the TA1 portion below the interface is computed within the black square in Figure 8(d1,e1); (**b**) stress analysis of the HDI in the vicinity of the MB interface with strain of 0.28.

Observing the MB’s interface edge position, see Figure 8(e1), the interface is found to be at 45° from the loading direction. Below the interface is the TA1 microstructure, in which the twins are also $\left\{10\bar{1}2\right\}$-type twins according to the analysis of the characteristic angles in Figure 8(e2). The average KAM values of TA15 above the interface and TA1 below the interface in Figure 8(e3) are calculated, and it is found that the average KAM value of TA1 below the interface is larger. It indicates that compared with the TA15 above the interface, the TA1 below the interface is more stress-concentrated and carries more stress during the dynamic deformation process. Figure 9a shows the twinning density of TA1 microstructures at different locations in Figure 8. It is found that the twinning density in the black box in Figure 8(e1) is smaller than that in Figure 8(d1). Still, the carrying stress in the black box in Figure 8(e1) is smaller than that in Figure 8(d1), which suggests that at the edge of the interface, there is a deformation mechanism of the TA1 microstructure in addition to twinning, and there is also a slip. This is due to the plastic incompatibility of the HS materials in the deformation process. In the TA1 part, GNDs are accumulated near the interface, and the density of GNDs increased in the locations closer to the edge of the interface. As consequence, there is a large coordinated deformation effect near the interface, and the interface at location e in Figure 8a was concave towards the TA1 part. Figure 9b shows the HDI stress analysis of the MB, which decomposes the forward and back stresses at the interface edge, and in addition to a pair of stresses in the vertical direction, there is also a pair of stresses in the horizontal direction, which has a tearing effect near the interface. With the increase in plastic strain, the back stress in the TA1 microstructure increases until cracks are produced at the interface edges, which is the reason for the sprouting of the ASB at the interface edges.

Observation of the microstructure of the MB near the surface of TA1, see Figure 8(f1,g1), reveals that a large number of twins exist within the microstructure of TA1, and according to the analysis of the characteristic corners in Figure 8(f2,g2), both $\left\{10\bar{1}2\right\}$-type tensile twins and $\left\{11\bar{2}2\right\}$-type compression twins exist, which suggests that the state of the force at each location near the surface of TA1 is similar. This is in contrast with Figure 8(d1,e1), where only $\left\{10\bar{1}2\right\}$-type tensile twins exist near the MB’s interface. The coordinated deformation effect between the HS materials near the interface is dominant and there is a tensile effect of the part of TA15 with a small deformation on the part of TA1 with a large deformation. The larger average value of KAM in Figure 8(f3,g3) indicates that there is a stress concentration in the MB near the surface location of TA1, which carries a larger stress during the dynamic deformation process, which coincides with Figure 4a in which the heterogeneous material carries a larger stress in the TA1 portion first at the early stage of dynamic deformation.

**Figure 10:**
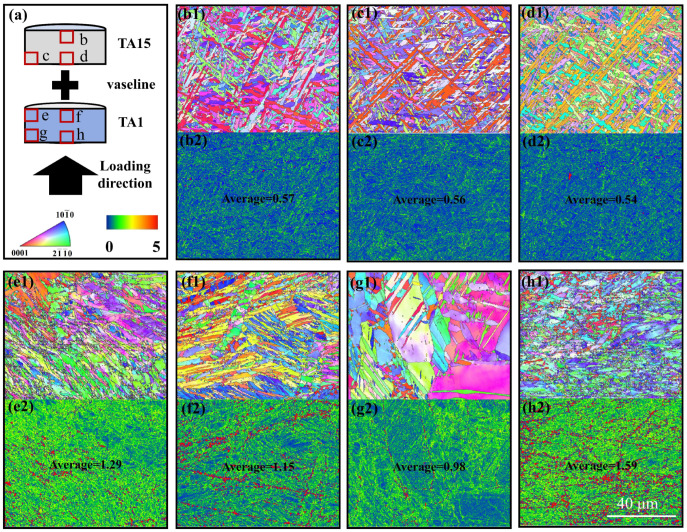
Grain orientation maps and dislocation density distribution cloud maps at various locations after dynamic deformation of MB with a strain of 0.28: (**a**) schematic diagram of NMB loading, with scales of polar and KAM maps of IPF; (**b1**,**c1**,**d1**,**e1**,**f1**,**g1**,**h1**) IPF maps at the red square position in schematic (**a**); (**b2**,**c2**,**d2**,**e2**,**f2**,**g2**,**h2**) are the red square location KAM plots, where the mean value of KAM has been calculated and is labeled in the KAM plots.

Figure 10 shows the specimen’s longitudinal profile EBSD results after dynamic NMB compression with a strain of 0.28, where Figure 10a shows the loading schematic for the NMB, and Figure 10(b1–h2) show the IPF and KAM plots at the red box position of the schematic. Observing the IPF and KAM plots of each part of the TA15 matrix in the NMB, see Figure 10(b1–d2), it is found that the average value of KAM is small, which indicates that the stress concentration of each part of TA15 is low, and the TA15 part of the NMB carries less stress.

Comparative analysis of the IPF maps of each part of the TA1 section in the NMB is shown in Figure 10(e1,f1,g1,h1), which shows that due to the large amount of deformation, there are more regions with lower recognition rates in the TA1 microstructure. There are more elongated grains along the 45° direction in the lower left corner and more twins in the upper right corner of Figure 10(e1), which will produce ASBs in the direction of grain elongation with the increase in strain, which is consistent with the cracking of the NMB at the edge of TA15 and the interface junction in Figure 7. Comparative analysis of Figure 10(e2,f2,g2,h2) reveals that Figure 10(h2) has the largest mean value of KAM, Figure 10(h2) has the largest mean value of KAM, Figure 10(e2) is the next largest, and Figure 10(g2) is the smallest, which indicates that the stress concentration is the largest in Figure 10(h2), and that it carries the largest amount of stress in the dynamic deformation process.

The MB is analyzed in comparison with the NMB. Regarding the TA15 portion, the TA15 portion near the MB interface also carries a large amount of stress, but the TA15 portion in the NMB carries almost no stress. Regarding the TA1 fraction, it was found that the TA1 matrix in the NMB carries more stress overall. Therefore, the NMB will experience damage earlier than the MB, with less energy absorbed before the material is damaged. However, compared with the MB, the stress at the h position of the NMB in Figure 10a is smaller, which is due to the uncoordinated deformation effect of the microstructures on both sides of the interface during the dynamic deformation process and the protruding portion at the edge of TA1 is not subjected to the stress of TA15.

## 4. Conclusions

This paper investigates the dynamic deformation behavior of TA1/TA15 3D-printed HS materials. The adiabatic shear sensitivity of heterogeneous titanium alloys with different interfacial bonding characteristics is evaluated using the energy absorbed by the samples before adiabatic shear damage occurs as a characterizing parameter, revealing the microstructure state and adiabatic shear failure mode of the HS materials under the conditions of uniaxial dynamic compression, with the following conclusions:

(1) Compared to the NMB, the MB has better resistance to adiabatic shear breakage and absorbs more energy per unit volume before damage occurs, a year-on-year improvement of about 12%.
(2) In both HS materials, the ASB is concentrated in the TA1 portion, sprouting from the edge of the interface, expanding in the 45° direction, and eventually forming a “V-shaped” crack.
(3) For MB, near the surface of TA15 carries less stress, both TA1 and TA15 microstructures near the interface carry more stress. There are two deformation mechanisms, slip and twinning, within the TA1 microstructure. The dislocation density at the interface is large, and slip is the main deformation mechanism; near the TA1 surface, twinning is the main deformation mechanism, and $\left\{10\bar{1}2\right\}$-type tensile twins and $\left\{11\bar{2}2\right\}$ type compression twins exist.
(4) Compared to the MB, the TA15 portion of the NMB carries almost no stress due to its poor ability to coordinate deformation near the interface, but the TA1 portion of the NMB carries more stress and thus will experience damage earlier than the MB.

## Figures and Tables

**Table 1:** Chemical composition analysis of 3D-printed TA15 and TA1 materials (wt.%).

	Ti	Al	Mo	V	Zr	Fe	N	C	H	O
TA15 (powder)	Bal.	6.56	1.92	2.32	2.26	0.048	0.010	0.019	0.0027	0.122
TA1 (powder)	Bal.	-	-	-	-	0.019	0.003	0.008	0.001	0.064
TA15 (printed)	Bal.	6.60	1.62	2.08	2.20	0.062	0.092	0.016	0.0024	0.12
TA1 (printed)	Bal.	-	-	-	-	0.024	0.0094	0.0086	<0.002	0.049

## Data Availability

Data are contained within the article.

## References

[1] Colorado H.A., Cardenas C.A., Gutierrez-Velazquez E.I., Escobedo J.P., Monteiro S.N. (2023). Additive manufacturing in armor and military applications: Research, materials, processing technologies, perspectives, and challenges. J. Mater. Res. Technol..

[2] Alaghmandfard R., Chalasani D., Odeshi A., Mohammadi M. (2021). Activated slip and twin systems in electron beam melted Ti-6Al-4V subjected to elevated and high strain rate dynamic deformations. Mater. Charact..

[3] Alaghmandfard R., Seraj P., Sanjari M., Pirgazi H., Dharmendra C., Odeshi A.G., Shalchi Amirkhiz B., Mohammadi M. (2022). High strain rate deformation behavior, texture and microstructural evolution, characterization of adiabatic shear bands, and constitutive models in electron beam melted Ti-6Al-4V under dynamic compression loadings. J. Mater. Res. Technol..

[4] Salehi S.-D., Beal R., Kingstedt O.T. (2023). Dynamic behavior and thermomechanical characterization of laser powder bed fusion and wrought Ti–6Al–4V. Int. J. Impact Eng..

[5] Awannegbe E., Chen L., Zhao Y., Qiu Z., Li H. (2024). Effect of thermomechanical processing on compressive mechanical properties of Ti–15Mo additively manufactured by laser metal deposition. Mater. Sci. Eng. A.

[6] Sukumar G., Patra A.K., Kumar A., Thakur R.K., Singh B.B., Bhattacharjee A., Sarma V.S. (2024). A comparative study on dynamic deformation and ballistic impact response of Ti–4Al–2.5V–1.5Fe–0.25O and Ti–6Al–4V alloys. Mater. Sci. Eng. A.

[7] Zhu Y., Wu X. (2023). Heterostructured materials. Prog. Mater. Sci..

[8] Ashby M.F. (2006). The deformation of plastically non-homogeneous materials. Philos. Mag. A J. Theor. Exp. Appl. Phys..

[9] Wang M., Guo F., He Q., Su W., Ran H., Cheng Q., Kim H.S., Wang Q., Huang C. (2023). Superior strength-ductility synergy by microstructural heterogeneities in pure titanium. Mater. Sci. Eng. A.

[10] Zhang D., Zhang M., Lin R., Liu G., Li J., Feng Y. (2021). Strengthening and strain hardening mechanisms of a plain medium carbon steel by multiscale lamellar structures. Mater. Sci. Eng. A.

[11] Liu X.L., Xue Q.Q., Wang W., Zhou L.L., Jiang P., Ma H.S., Yuan F.P., Wei Y.G., Wu X.L. (2019). Back-stress-induced strengthening and strain hardening in dual-phase steel. Materialia.

[12] Bhagirath Jadhav A., Gaikwad A., Gori Y., Somaiah A., Rambabu G.V., Al-Ataby F.H., Saxena K.K. (2023). A review of armour’s use of composite materials. Mater. Today Proc..

[13] Wu H., Fan G. (2020). An overview of tailoring strain delocalization for strength-ductility synergy. Prog. Mater. Sci..

[14] Daram P., Hiroto T., Watanabe M. (2023). Microstructure and phase evolution of functionally graded multi-materials of Ni–Ti alloy fabricated by laser powder bed fusion process. J. Mater. Res. Technol..

[15] Dixit S., Dash B.B., Kumar D., Bhattacharjee A., Sankaran S. (2023). Influence of phase morphology, static recrystallization, and crystallographic texture on room temperature tensile properties of Ti–6Al–4V alloy: Comparison between post-tested equiaxed, bimodal, and lamellar microstructures. Mater. Sci. Eng. A.

[16] Ji W., Zhou R., Vivegananthan P., See Wu M., Gao H., Zhou K. (2023). Recent progress in gradient-structured metals and alloys. Prog. Mater. Sci..

[17] Park C.W., Hajra R.N., Kim S.H., Lee S.-H., Kim J.H. (2023). Optimizing multi-interlayered additive manufacturing for high strength robust joints in Inconel 718 and Ti–6Al–4V alloys. J. Mater. Res. Technol..

[18] Jeong H.-I., Kim D.-H., Lee C.-M. (2024). Multi-material deposition of Inconel 718 and Ti–6Al–4V using the Ti–Nb–Cr–V–Ni high entropy alloy intermediate layer. J. Mater. Res. Technol..

[19] Huang C.X., Wang Y.F., Ma X.L., Yin S., Höppel H.W., Göken M., Wu X.L., Gao H.J., Zhu Y.T. (2018). Interface affected zone for optimal strength and ductility in heterogeneous laminate. Mater. Today.

[20] Wu H., Huang M., Xia Y., Li X., Li R., Liu C., Gan W., Xiao T., Geng L., Liu Q. (2023). The importance of interfacial stress-affected zone in evading the strength-ductility trade-off of heterogeneous multi-layered composites. Int. J. Plast..

[21] Lu X., Zhao J., Wang Q., Ran H., Wang Q., Huang C. (2023). Effect of stress/strain partition on the mechanical behavior of heterostructured laminates: A strain gradient plasticity modeling. Results Eng..

[22] Zhuo X., Gao W., Zhao L., Zhao S., Liu H., Hu Z., Zhang P., Wu Y., Jiang J., Ma A. (2023). A bimodal grain structured Zn-0.4Mg-0.02Mn alloy with superior strength-ductility synergy. Mater. Sci. Eng. A.

[23] Hua A., Su Y., Cai Y., Cao H., Liu K., Guan L., Zhang D., Ouyang Q. (2023). Balanced strength and ductility in B4C/SiC/2024Al composite with multimodal grain structure achieved by multistep ball milling and powder metallurgy. Mater. Charact..

[24] Yu Y., Wang X., Wu Y., Ma M., Gao G. (2024). The impact of backplate support conditions on ceramic fracture and energy absorption in the penetration resistance process of ceramic/metal composite armor. Ceram. Int..

[25] Pan G., Su H., Li X., Wang J. (2023). Coupled FEM-SPH simulation of the protective properties for metal/ceramic composite armor. Int. J. Lightweight Mater. Manuf..

[26] Miao Y., Yuan M., Fan Z., Wang X., Li Z., Zhou X., Wang H. (2022). Effect of hot-pressing temperature on microstructure and the improvement of residual Al on tensile ductility of Ti/Al3Ti heterogeneous structure. Mater. Sci. Eng. A.

[27] Fan W., Dong L., Zheng F., Li M., Sun G., Xu J., Li X., Fu Y., Elmarakbi A., Wang L. (2023). Heterogeneous network structures formed in TiC/TC4/β-Ti composites for enhancing strength with good ductility. Compos. Commun..

[28] Jiang P.F., Nie M.H., Teng J.Z., Liu C.Z., Zhang Z.H. (2023). Multi-wire arc additive manufacturing of TC4-Nb-NiTi bionic layered heterogeneous alloy: Microstructure evolution and mechanical properties. Mater. Charact..

[29] Shakil M., Ahmad M., Tariq N.H., Hasan B.A., Akhter J.I., Ahmed E., Mehmood M., Choudhry M.A., Iqbal M. (2014). Microstructure and hardness studies of electron beam welded Inconel 625 and stainless steel 304L. Vacuum.

[30] Carroll B.E., Otis R.A., Borgonia J.P., Suh J.-o., Dillon R.P., Shapiro A.A., Hofmann D.C., Liu Z.-K., Beese A.M. (2016). Functionally graded material of 304L stainless steel and inconel 625 fabricated by directed energy deposition: Characterization and thermodynamic modeling. Acta Mater..

[31] Liu Y., Zhang Y. (2020). Microstructure and mechanical properties of TA15-Ti2AlNb bimetallic structures by laser additive manufacturing. Mater. Sci. Eng. A.

[32] Wang H., Ma S.-Y., Wang J.-C., Lu T., Liu C.-M. (2021). Microstructure and mechanical properties of TA15/TC11 graded structural material by wire arc additive manufacturing process. Trans. Nonferrous Met. Soc. China.

[33] Pacheco J.T., Meura V.H., Bloemer P.R.A., Veiga M.T., de Moura Filho O.C., Cunha A., Teixeira M.F. (2022). Laser directed energy deposition of AISI 316L stainless steel: The effect of build direction on mechanical properties in as-built and heat-treated conditions. Adv. Ind. Manuf. Eng..

[34] Zhang F., Shen Y., Yang K., Ma X., Yang H. (2024). Microstructure and hardness of S304/Ni25/TC4 functionally graded materials fabricated by laser solid forming. Vacuum.

[35] He J., Yuan F., Yang M., Jiao S., Wu X. (2019). Superior mechanical properties and deformation mechanisms of heterogeneous laminates under dynamic shear loading. Mater. Sci. Eng. A.

[36] Guo Y.-s., Chen P.-w., Arab A., Zhou Q., Mahmood Y. (2020). High strain rate deformation of explosion-welded Ti6Al4V/pure titanium. Def. Technol..

[37] Liang Y.-J., Liu D., Wang H.-M. (2014). Microstructure and mechanical behavior of commercial purity Ti/Ti–6Al–2Zr–1Mo–1V structurally graded material fabricated by laser additive manufacturing. Scr. Mater..

[38] Fang X.T., He G.Z., Zheng C., Ma X.L., Kaoumi D., Li Y.S., Zhu Y.T. (2020). Effect of heterostructure and hetero-deformation induced hardening on the strength and ductility of brass. Acta Mater..

[39] Kennedy J.R., Davis A.E., Caballero A.E., White M., Fellowes J., Pickering E.J., Prangnell P.B. (2021). Microstructure transition gradients in titanium dissimilar alloy (Ti-5Al-5V-5Mo-3Cr/Ti-6Al-4V) tailored wire-arc additively manufactured components. Mater. Charact..

[40] Jiang P.F., Nie M.H., Zong X.M., Wang X.B., Chen Z.K., Liu C.Z., Teng J.Z., Zhang Z.H. (2023). Microstructure and mechanical properties of TC4/NiTi bionic gradient heterogeneous alloy prepared by multi-wire arc additive manufacturing. Mater. Sci. Eng. A.

